# An unusual case of splenic B‐cell lymphoma/leukemia

**DOI:** 10.1002/jha2.164

**Published:** 2021-02-16

**Authors:** Mehrnoosh Tashakori, Pei Lin

**Affiliations:** ^1^ Department of Hematopathology The University of Texas MD Anderson Cancer Center Houston Texas

A 76‐year‐old man with a history of relapsed/refractory splenic marginal zone lymphoma (SMZL) underwent splenectomy for cytoreduction (WBC 133.7 10^9^/L) after failing rituximab, ibrutinib, bendamustine, obinutuzumab, and cyclophosphamide. The spleen (3470 grams) had a homogeneous cut surface on gross examination (Panel A). Microscopically, the infiltrate is composed of small to medium sized lymphoid cells involving chiefly the red pulp. (Panel B: Diff‐Quik stain, original magnification ×1000; Panel C‐D: H&E, original magnification ×20 and ×400, respectively).

Flow cytometry detected monotypic B‐cells positive for CD5 (partial), CD103 (dim), and negative for CD25 and CD200. By immunohistochemistry, the lymphoma cells were positive for PAX‐5, CD5 (partial/weak) (Panel E: PAX‐5/CD5 dual stain, original magnification ×400), IgD (Panel F, original magnification ×400), DBA.44 (Panel G, original magnification ×400), and negative for Cyclin D1, SOX11, IgG, and BRAF V600E. The proliferation rate was approximately 50% (Panel H, Ki67 stain, original magnification ×400). Karyotyping showed complex aberrations including i(17q). FISH studies confirmed*TP53* deletion (Panel I). Next generation sequencing revealed *TP53* E56*, a nonsense mutation in transactivation domain (TAD), and no *BRAF*V600E or *MAP2K1* mutations. The patient passed away shortly after splenectomy.

**FIGURE 1:**
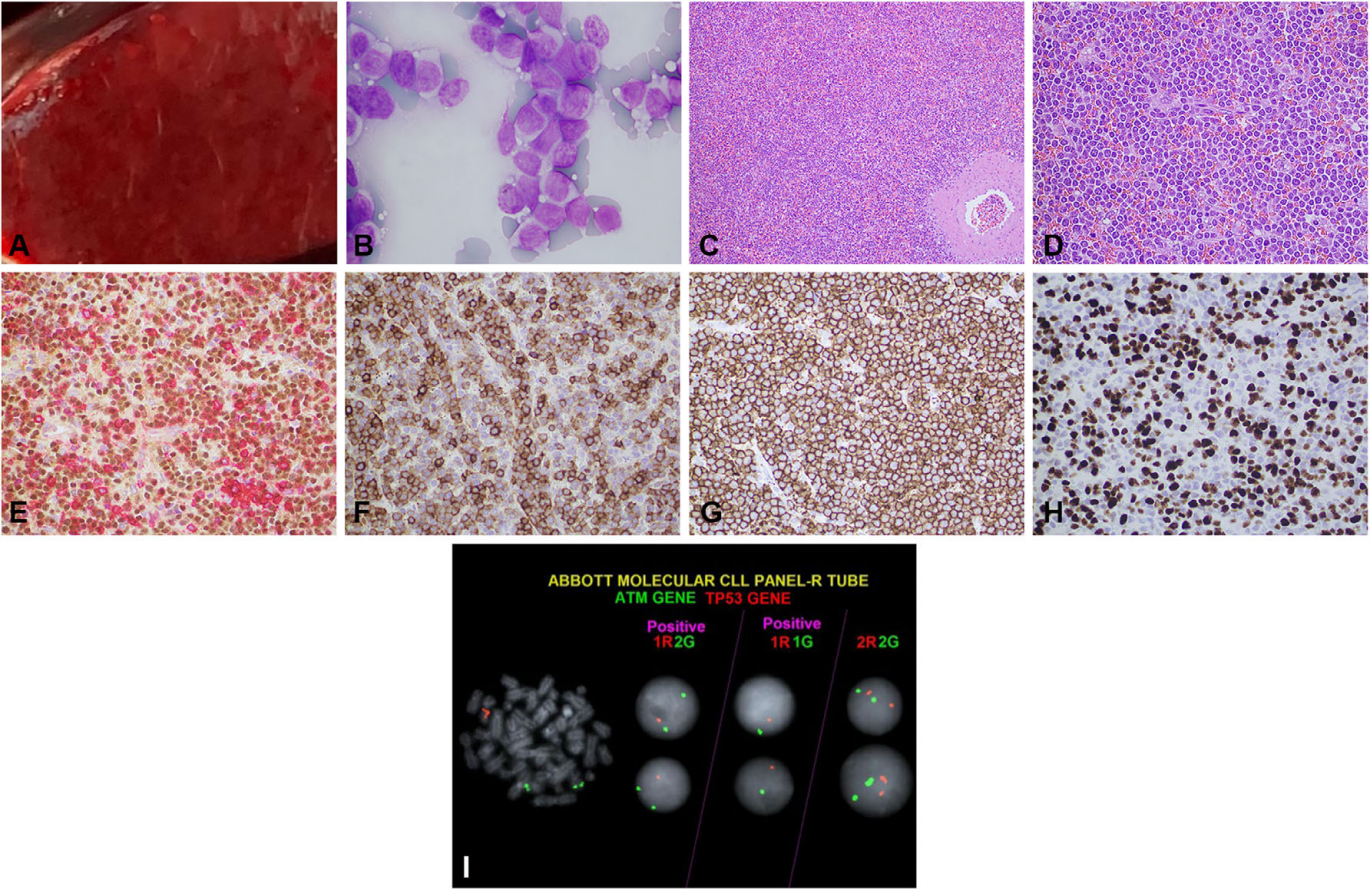


Though originally diagnosed with SMZL, the diffuse red pulp involvement in the case favors more a diagnosis of splenic red pulp lymphoma/leukemia, a provisional entity in the 2017 World Health Organization classification characterized by proliferation of predominately small mature B cells and an indolent clinical course. The highly aggressive clinical course and the unusual morphology (increased medium sized, high Ki67) in the current case are most likely related to a “null” *TP53* status, an early nonsense mutation in TAD in one allele and deletion of another allele, that is, bi‐allelic inactivation of *TP53* gene. The case also illustrates the diagnostic challenge in distinguishing SMZL from splenic red pulp lymphoma/leukemia in the absence of a splenectomy specimen as the two share common immunophenotypic profile.

